# Potential benefits and risks of solar photovoltaic power plants on arid and semi-arid ecosystems: an assessment of soil microbial and plant communities

**DOI:** 10.3389/fmicb.2023.1190650

**Published:** 2023-08-01

**Authors:** Ziyu Liu, Tong Peng, Shaolan Ma, Chang Qi, Yanfang Song, Chuanji Zhang, Kaile Li, Na Gao, Meiyun Pu, Xiaomin Wang, Yurong Bi, Xiaofan Na

**Affiliations:** ^1^Key Laboratory of Cell Activities and Stress Adaptations, Ministry of Education, School of Life Sciences, Lanzhou University, Lanzhou, China; ^2^No. 1 Middle School of Penyang, Guyuan, China

**Keywords:** arid ecosystem, fungi, photovoltaic panels, plant communities, precipitation, prokaryotes, shade, soil

## Abstract

Exponential increase in photovoltaic installations arouses concerns regarding the impacts of large-scale solar power plants on dryland ecosystems. While the effects of photovoltaic panels on soil moisture content and plant biomass in arid ecosystems have been recognized, little is known about their influence on soil microbial communities. Here, we employed a combination of quantitative PCR, high-throughput sequencing, and soil property analysis to investigate the responses of soil microbial communities to solar panel installation. We also report on the responses of plant communities within the same solar farm. Our findings showed that soil microbial communities responded differently to the shading and precipitation-alternation effects of the photovoltaic panels in an arid ecosystem. By redirecting rainwater to the lower side, photovoltaic panels stimulated vegetation biomass and soil total organic carbon content in the middle and in front of the panels, positively contributing to carbon storage. The shade provided by the panels promoted the co-occurrence of soil microbes but inhibited the abundance of *16S rRNA* gene in the soil. Increase in precipitation reduced *18S rRNA* gene abundance, whereas decrease in precipitation led to decline in plant aboveground biomass, soil prokaryotic community alpha diversity, and dehydrogenase activity under the panels. These findings highlight the crucial role of precipitation in maintaining plant and soil microbial diversities in dryland ecosystems and are essential for estimating the potential risks of large-scale solar power plants on local and global climate change in the long term.

## Introduction

As alternatives to powerplants based on fossil fuels, solar photovoltaic power plants have become increasingly eminent energy sources. Coupled with declines in the prices of solar photovoltaic panels, the requirement for clean energy exponentially boosted the construction of photovoltaic power stations in recent decades in Asia, specifically in the arid and semi-arid regions of northwest China. With abundant solar irradiation (5400–6700 MJ/m^2^·yr^−1^, Sun et al., 2014) and vast suitable land resources, northwest China had a total installed photovoltaic capacity of 48,330 MW in 2019 (Xia et al., 2022) and still has enormous potential to continue growing (Qiu et al., 2022). The development of power plants in northwest China has positively contributed to carbon emission reduction, with a total net carbon reduction of 23.27 × 10^9^ kg carbon dioxide from 2009 to 2017 (Pu et al., 2021). However, given that the installation and operation processes of large-scale soil power plants lead to local climate changes (Barron-Gafford et al., 2016; Yang et al., 2017; Li et al., 2018), ecological issues concerning the potential impacts of the power plants on the fragile ecosystems of the arid and semi-arid regions have attracted considerable attention (Turney and Fthenakis, 2011; Szilágyi and Gróf, 2020; Yue et al., 2021; Wu et al., 2022).

Apart from the alternation of air temperature, relative humidity, and wind speed and direction, the most significant impacts of solar photovoltaic panels on the microenvironments of dryland ecosystems are the stimulation of surface soil moisture and decreased soil temperature due to the shadows cast by the panels (Hassanpour Adeh et al., 2018; Yue et al., 2021; Wu et al., 2022). Such shifts decrease the rate of water loss by inhibiting evapotranspiration, leading to increased water availability for plants in the entire growing season and indirectly promoting vegetation biomass and coverage (Hassanpour Adeh et al., 2018; Liu et al., 2019). Additionally, the alternations in microenvironments have been found to enhance the survival of plant species’ seed bank in shaded areas (Hernandez et al., 2020). Furthermore, Li et al. (2018) demonstrated that the implementation of large-scale wind and solar farm in the Sahara could induce localized rainfall, particularly in the nearby Sahel region. This effect is attributed to increased surface drag, reduced albedo, and subsequent vegetation expansion, creating a positive feedback loop for increased precipitation. Consequently, the installation of solar power plant facilities has been regarded as a “win-win” strategy, as it simultaneously reduces carbon emissions and prevents desertification in arid regions (Li et al., 2018; Liu et al., 2019, 2020).

However, recent experimental evidence has shown that the annual mean temperature over a solar power plant is 2.4°C above that of a nearby desert region in an entire year (Barron-Gafford et al., 2016). The heat island effect of a large-scale photovoltaic installation might disrupt wildlife, habitat, and human health (Coutts et al., 2013; He et al., 2022). For instance, a 14-month study conducted in the Mojave Desert revealed a minimal but negative impact on the bird life near solar power plants (McCrary et al., 1984). Moreover, the movement of wild animals is limited by fences around power plants (Turney and Fthenakis, 2011). Some studies have recommended the comprehensive evaluation of the ecological effects of large-scale photovoltaic installations from the perspectives of different biological populations in representative ecosystems. Currently, few studies have focused on the responses of soil microbial populations to solar power plants (Yuan et al., 2022).

Previous studies have demonstrated the sensitivity of soil microbial communities in dryland ecosystems to multiple environmental factors, including soil moisture availability and temperature (Bell et al., 2008), precipitation patterns (Nielsen and Ball, 2015; Na et al., 2019a), as well as the presence and composition of vegetation (Strukelj et al., 2021). Increase in soil moisture content can alter microbial community composition (i.e., increase the ratio between fungi and bacteria and inhibit the proliferation of Actinobacteria; Bell et al., 2014) and regulate soil microbial biomass, respiration, and metabolic activity (Banerjee et al., 2016). Owing to increased resource availability (i.e., organic carbon and nitrogen) and the limited diffusion of gases (i.e., oxygen and methane), increase in soil moisture activates microbial respiration (Liu et al., 2009) and N_2_O emission by up-regulating the expression of ammonia monooxygenase and nitric oxide reductase genes (Banerjee et al., 2016). By contrast, increase in soil temperature reduces soil water availability and negatively regulates the physiological activities of soil microbes (Liu et al., 2009). The alternation of precipitation regimes affects the diversity and functionality of soil microorganisms by controlling soil water content and plant biomass (Na et al., 2019a). Under increasing precipitation conditions, promoted plant growth increases organic carbon inputs, thus leading to changes in soil bacterial community composition in arid ecosystems (Nielsen and Ball, 2015; Na et al., 2019a).

Given the impacts of photovoltaic panels on soil moisture, temperature (Yue et al., 2021; Wu et al., 2022), and vegetation (Liu et al., 2019), large-scale photovoltaic installation is expected to fundamentally alter the diversity, activity, and functionality of soil microbial communities. However, questions about the responses of soil microorganisms to photovoltaic installations and the potential influences of the responses on dryland ecosystems are yet to be confirmed experimentally. To answer these questions, we conducted a field experiment in a solar photovoltaic power plant established in 2016 in northwest China. By combining barcode sequencing, real-time PCR, enzymatic assay, and soil abiotic property analysis, we evaluated how photovoltaic panels affect plant richness and biomass, soil abiotic properties, and soil prokaryotic and fungal community diversity, abundance, and activities in different zones underneath and in front of the panels. Specifically, the present study tested the following hypotheses: (1) the presence of solar photovoltaic panels indirectly modifies diversity and activity of soil microbial community through alterations in plant and soil properties in different areas within the solar farm; and (2) the modulation of precipitation amount by the solar panel can further modify the shading effect on soil microbial community. By investigating these hypotheses, this study aimed to contribute to the understanding of the ecological impacts of large-scale solar panel installation on plant and soil microbial communities in arid and semi-arid ecosystems.

## Materials and methods

### Research site

The solar power plant is located in Hongsipu District, Ningxia Hui Autonomous Region, northwest China (37° 36′ 47′′ N; 106° 7′ 40′′ E). Spanning across an expansive area of approximately 700 hectares, the power plant region belongs to a classical temperate continental and arid monsoonal climate zone with a mean annual temperature of 10.6°C and mean annual precipitation of 197.9 mm. A considerable amount of rainwater (>85%) is deposited during the growing season from April to September. The habitat variety of the study site is desert steppe, and the primary soil type is sandy loam. Since the establishment of the power plant in 2016, the area has been isolated by fencing, thereby preventing interference by human activities (i.e., grazing).

### Sampling

**Figure 1:**
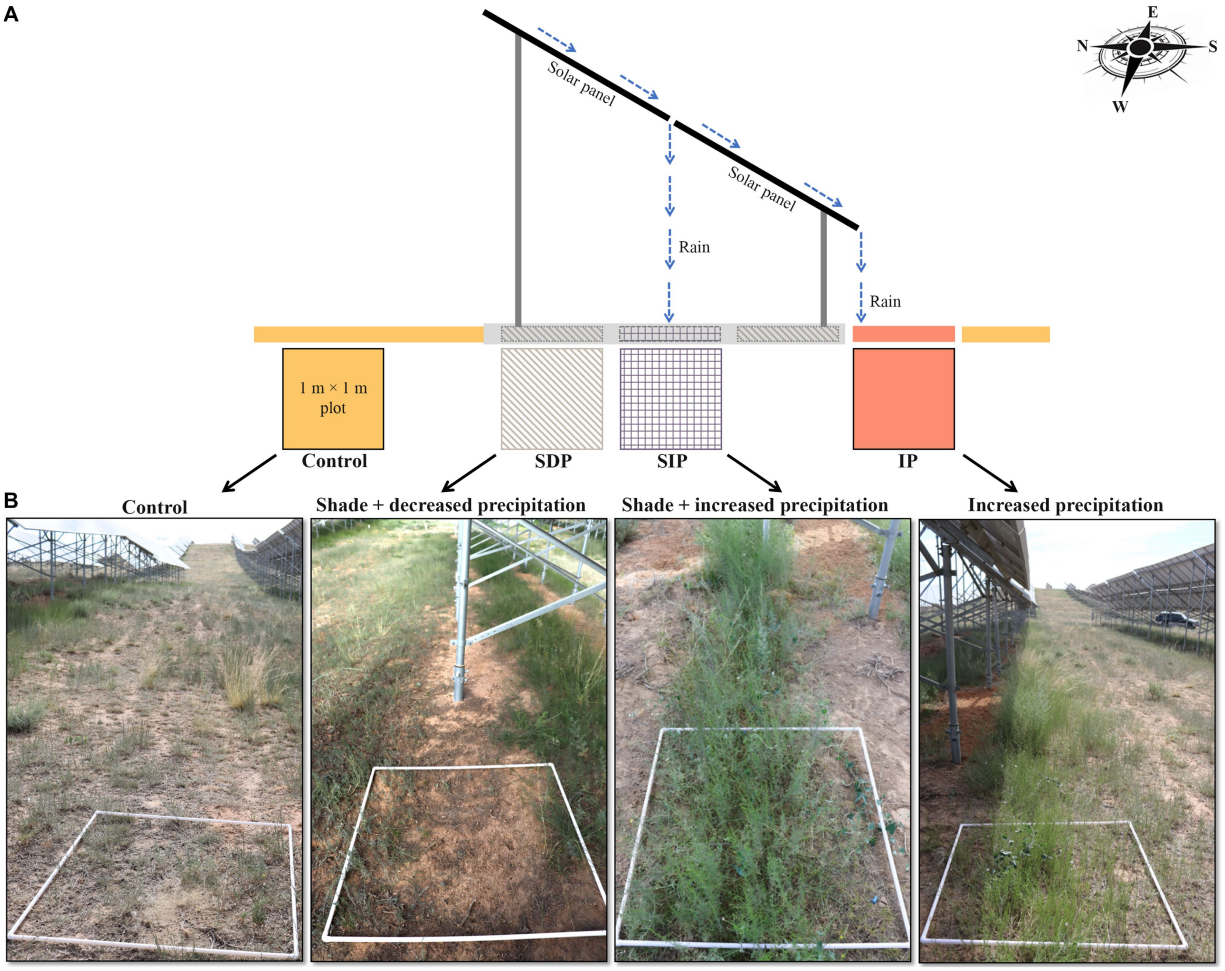
Variations in vegetations and microenvironments under and in front of solar photovoltaic panels within the solar farm. **(A)** Specification for the categories of microenvironments modified by the different effects of photovoltaic panels. The tilt angle of the panel was 37.5°. The width and length of the sub-panel were 95 and 185 cm, respectively. The gap between the upper and lower sub-panel was 5 cm. **(B)** Details of each sampling plot studied in the present study.

The field experiment was conducted in July of 2021, encompassing a total of five sites chosen within the solar farm to ensure similar conditions and a minimum distance of 3 km between each site (*n* = 5). To mitigate potential influence of slope on the analysis of plant and soil microbial communities, flat areas were selected at each sampling site. These areas were oriented parallel to the alignment of the photovoltaic panels, spanning approximately 50 m in length from east to west. Each of these areas was further divided into three distinct treatments, namely, shade and decreased precipitation treatment (referred to as SDP), shade and increased precipitation treatment (SIP), and increased precipitation treatment (IP), taken into account variations in shade and water availability (see Figure 1 for detailed treatment descriptions). Additionally, an area located in the middle of two solar panel rows was designated as the control. Each treatment category consisted of a 1 m × 1 m plot, ensuring a minimum buffer zone of 5 m between neighboring plots. Consequently, a total of 20 independent plots (four treatments × five replicates) were investigated in the present study. Considering the potential indirect effects of solar panels on the area between solar panel rows where the control was positioned, the comparisons conducted in this study generally provide insights into the specific impacts of solar panels within the solar farm.

Plants were cut off at the soil surface and categorized according to species in each plot. After the entire plant shoot was trimmed, five soil cores were randomly sampled in each plot using a soil auger (20 cm in depth; 5 cm in diameter) and then pooled into a mixed sample. All sorted plant shoots and soil samples were placed in a cool sampling box (~10°C) and returned to the laboratory. In the laboratory, some of the soil samples (without plant debris and gravel) were directly weighted and then oven-dried at 105°C for 24 h to determine soil gravimetric water content. All belowground plant tissues were then carefully collected by sieving the soil samples at 2 mm. After being thoroughly homogenized, half of the sieved soils were air-dried for soil abiotic property determination, whereas the rest were used for enzymatic activity assay and DNA isolation immediately. The plant aboveground and belowground biomasses were assayed after drying at 75°C for 48 h. Plant community richness was calculated according to the number of individual plant species in each plot, and the plant community Shannon index was estimated by using the following function: *H* = −Σpi*ln(*pi*), where *pi* is the proportion of the entire community’s aboveground biomass made up by individual plant species *i*.

### Soil abiotic property detection

Soil organic carbon and total nitrogen were determined using an elemental analyzer (vario EL cube, Elementar, Berlin, Germany). Soil total phosphorus content was detected by vanadium molybdate yellow colorimetry (Na et al., 2019b). Dissolved organic carbon and nitrogen were extracted with 0.5 M K_2_SO_4_ and then assayed using a total organic carbon analyzer (vario TOC cube, Elementar, Berlin, Germany). Available phosphorus was extracted with 0.5 M NaHCO_3_ and quantified using the method described by Olsen et al. (1954). Soil pH and electrical conductivity were estimated in a suspension of soil and water (1:5, W/V) by using a pH and conductivity meter, respectively.

### Soil enzyme activity assay

The activity of soil dehydrogenase was measured according to the reduction of 2,3,5-triphenyl tetrazolium chloride to 2,3,5-triphenyl formazan in a Tris buffer (pH = 7.6), as described by Kumar et al. (2013). Soil invertase was monitored by determining the production of reducing glucose after 24 h of incubation soil with buffered sucrose solution (pH = 5.5), as described by Frankeberger and Johanson (1983). Protease activity was detected by incubating soil samples with a buffered casein solution (pH = 7.6), as reported by Kandeler et al. (1999). The determination of acid phosphate activity utilized *p*-nitrophenyl phosphate disodium salt hexahydrate colorimetry according to the description by Dick (2011). The results of all the enzymes were expressed as μg h^−1^ g^−1^ dry weight of fresh soil.

### Soil DNA isolation and *16S*/*18S rRNA* gene quantification

The total genomic DNA of soil samples were extracted using a PowerSoil DNA isolation kit according to the manufacturer’s instructions (Qiagen, Hilden, Germany). Half of the high-quality genomic DNA samples were used for quantitative PCR assay, and the absolute abundance of *16S rRNA* and *18S rRNA* genes was determined. Specific primer sets Eub338 (5′-ACTCCTACGGGAGGCAGCAG-3′) and Eub518 (5′-ATTACCGCGGCTGCTGG-3′) were used in quantifying the abundance of *16S rRNA* (Fierer et al., 2005), whereas Fu18s1 (5′-GGAAACTCAGGTCCAGA-3′) and Nu-SSU-1536 (5′-ATTGCAATGCYCTATCCCCA-3′) were used in detecting the abundance of the *18S rRNA* in the soil (Borneman and Hartin, 2000). Each DNA sample was determined in triplicate on a QuantStudioTM 5 real-Time PCR system (Applied Biosystems, CA, USA) by using a TB green Premix Ex Taq kit. A real-time PCR reaction was performed as described in our previous study (Na et al., 2019b). A standard curve was generated using serial dilutions of the plasmid DNA containing the *16S rRNA* or *18S rRNA* gene fragment (*R*^2^ > 0.970, *p* < 0.001 in both cases).

### Amplicon sequence analysis

The other half of genomic DNA samples were used for amplicon sequencing. The 16S rRNA v4 region was amplified using the primer set 515F (5′-GTGCCAGCMGCCGCGGTAA-3′) and 806R (5′-GGACTACHVGGGTWTCTAAT-3′), while the ITS2 region was amplified by the primer pair ITS3-2024F (5′-GCATCGATGAAGAACGCAGC-3′) and ITS4-2409R (5′-TCCTCCGCTTATTGATATGC-3′) with attached sample-specific 6 bp barcodes. PCR amplification and sequencing library construction were performed as previously described (Na et al., 2019b). Sequencing was accomplished on an Illumina NovaSeq 6000 platform (Illumina, San Diego, CA, USA).

After raw reads were assigned to each sample by using unique barcodes, the sequences of the barcodes and primers were trimmed, and the reads were then merged by using FLASH (v1.2.11) according to the protocols described by Magoč and Salzberg (2011). Low-quality and chimeric sequences were filtered by using Qiime (v1.9.1; Caporaso et al., 2010) and UCHIME algorithm (v4.2; Edgar, 2013), respectively, prior to the clustering of qualified reads into operational taxonomic units (OTUs) at 97% sequence similarity. Taxonomic information for the OTUs was obtained via aligning the representative prokaryotic and fungal OTU reads in the SILVA rRNA database (v138.1; Quast et al., 2012) and the Unite database (v8.2; Kõljalg et al., 2013), respectively. On average, the sequencing resulted in 59,123 and 65,178 quality reads per sample, respectively, which assigned to 2,815 prokaryotic and 906 fungal OTUs (Supplementary Table S1). Before analyzing soil microbial community composition, we rarefied the OTU data to the sample with the minimum number of quality reads (55,256 for prokaryote and 47,478 for fungus). We further generated the rarefaction curves of the prokaryotic and fungal OTU numbers to determine whether the sequencing depth saturated under normalized read number (Supplementary Figure S1). Finally, the alpha diversity indices of soil microbial communities, including OTU richness and Shannon index, were calculated as described by a previous study (Wang et al., 2022). Sequencing data from the present work have been deposited with the NCBI Sequence Read Archive under BioProject: PRJNA883931.

### Statistical analysis

One-way ANOVA followed by Tukey HSD test was used in checking the statistical significance of variations in soil property, gene abundance, plant biomass, and the alpha diversity indices of soil microbial and plant communities among the treatments. The normality of the data was determined by using the Shapiro–Wilk test. To compare the mean differences in relative abundance data (i.e., composition of microbes and plant species) among treatments, we performed the Kruskal–Wallis test followed by a Mann–Whitney *U* test. Changes in soil microbial community composition at the OTU level were ordinated using principal coordinates analysis based on Bray–Curtis distance, and the factor significance of treatment was estimated using Adonis or pairwise Adonis test in the *vegan* R package. Before the analysis, rare OTUs (total number of <552 for prokaryotes and total number of <474 for fungi in all samples; *n* = 20) were removed, and the relative abundance data were Hellinger transformed. The independent and interactive effects of distinct data sets on shift in soil microbial community composition were determined through variance partitioning and distance-based RDA analysis. The factors included in each data collection were firstly chosen using a forward selection method.

To discover the interrelationships among treatments (photovoltaic panel), plant community, soil property, and microbial diversity, we constructed a structural equation model with Amos (IBM, USA) as we did in our previous study (Na et al., 2019b). We developed a prior model according to the results of variance partitioning analysis and then fitted our data to the model through maximum likelihood estimation. To evaluate the effects of the photovoltaic panels on microbial co-occurrence patterns, we analyzed two types of co-occurrence networks by using SparCC with default parameters. Bootstraps with 100 iterations were used in inferring pseudo *p* values. In both networks, the edges have correlation scores of >0.7 and < −0.7 were kept (*p* < 0.01). To explore the distribution patterns of treatment-specific OTUs in microbial co-occurrence patterns, we identified treatment-specific OTUs by combining the results of indicator species analysis (the *indicspecies* package) and likelihood ratio tests (the *edgeR* package) in R, as described by Hartman et al. (2018). The treatment-specific OTUs were then displayed in network modules by using the *igraph* R package.

## Results

### Responses of plants to the establishment of solar photovoltaic installations

**Figure 2:**
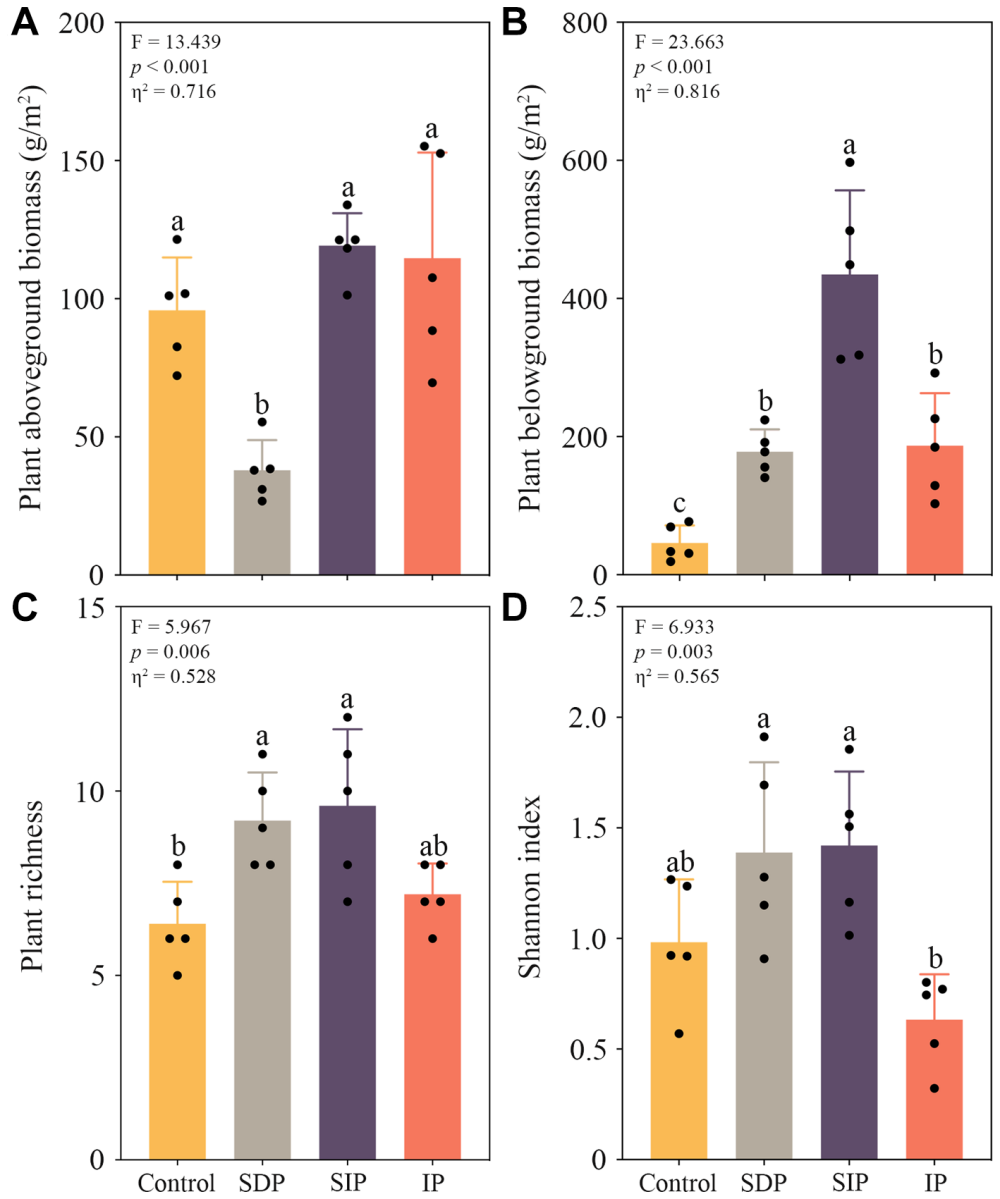
Responses of plant biomass and community alpha diversity to solar photovoltaic installations. **(A)** Shifts in plant aboveground and **(B)** belowground biomass in distinct treatments. **(C)** Variations in richness and **(D)** Shannon index of plant community among treatments. One-way ANOVA followed by the Tukey HSD test was used in determining statistically significant differences among treatments. Different lowercase letters indicated significant differences at the *p* < 0.05 level. Eta squared indicates the effect size of the treatment.

We observed a significant decrease in plant aboveground biomass in the treatment of SDP, whereas all treatments showed increases in belowground biomass (*p* < 0.05 in all cases, Figure 2). Specifically, the plant aboveground biomass in the SDP treatment was only 39.6% of that in the control (Figure 2A). In contrast, the belowground biomass in the SDP, SIP, and IP treatments increased by approximately 3.8, 9.5, and 4.1 times, respectively (Figure 2B). Photovoltaic panels stimulated the richness (*p* < 0.05, Figure 2C) and Shannon index of plant community (*p* > 0.05, Figure 2D) underneath. Four dominant plant species, namely, *Artemisia scoparia*, *Agropyron mongolicum*, *Rtemisia frigida*, and *Stipa capillata*, were observed in the dryland region. Of these plant species, the relative abundance of *Agropyron mongolicum* (Kruskal–Wallis *H* test, *H* = 8.188, *p* = 0.042) was inhibited, whereas those of the *Rtemisia frigida* (*H* = 9.024, *p* = 0.029) and other plant species (*H* = 8.291, *p* = 0.040, Supplementary Table S2) were stimulated by solar panels (Supplementary Table S2).

### Impacts of solar photovoltaic installations on soil properties

**Figure 3:**
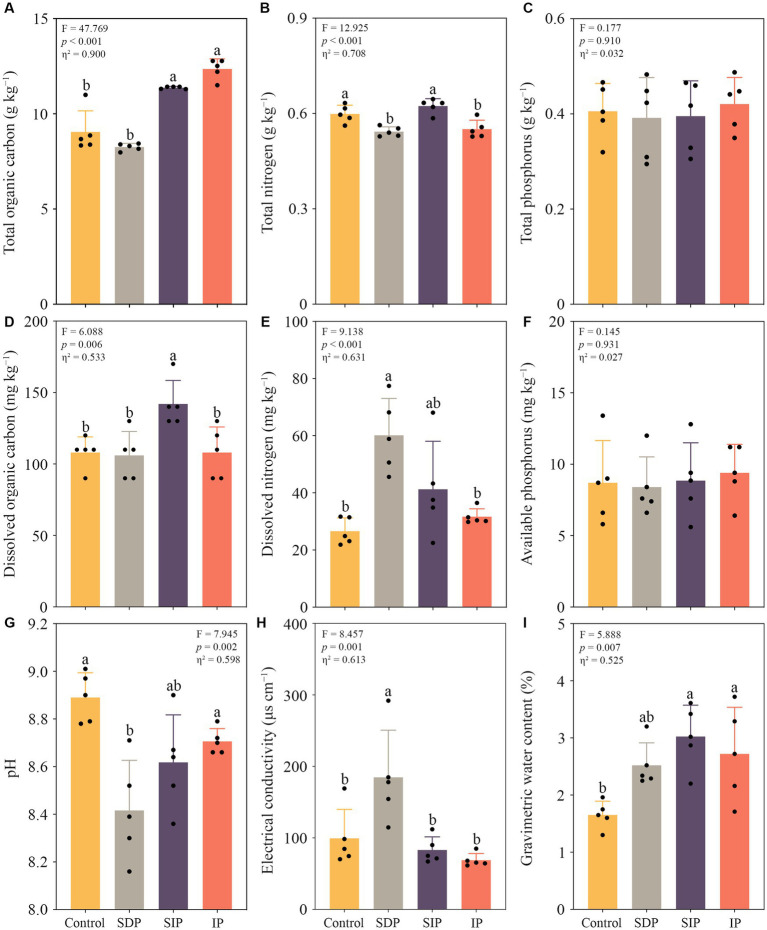
Impacts of solar photovoltaic installations on soil abiotic properties in arid and semi-arid ecosystems. **(A)** Variations in the total organic carbon, **(B)** total nitrogen, and **(C)** total phosphorus content within the soils under different treatments. **(D)** Alternations in the levels of dissolved organic carbon, **(E)** dissovled nitrogen concentration, and **(F)** available phosphorus concentration within the soils under distinct treatments. **(G)** Changes in soil pH, **(H)** electrical conductivity, and (I) gravimetric water content across the different treatments. Different lowercase letters indicated significant differences at the *p* < 0.05 level. Eta squared indicated the effect size of the treatment.

The photovoltaic installations significantly altered the total organic carbon and nitrogen content, dissolved organic carbon and nitrogen concentration, pH, electrical conductivity and the gravimetric water content of the surface soil (*p* < 0.01 in all cases, Figure 3). The total soil organic carbon content in IP and the total and dissolved organic carbon in SIP significantly increased compared with those in the control fields (*p* < 0.01, Figure 3). The total nitrogen content showed a slight decrease in both the SDP and IP treatments compared to the control and SIP treatments. Notably, there were no significant changes observed in the total and available phosphorus content across all treatments (Figure 3). In the treatment of SDP, the dissolved nitrogen concentration and soil electrical conductivity increased by 2.3- and 3.4-fold, respectively, whereas soil pH declined by an average of 0.47 unit relative to that in the control (*p* < 0.01, Figure 3). Similarly, the activity of soil dehydrogenase in the SDP treatment was inhibited by the photovoltaic panels (*p* < 0.05, Supplementary Figure S2).

### Responses of soil microbes to solar power plant

**Figure 4:**
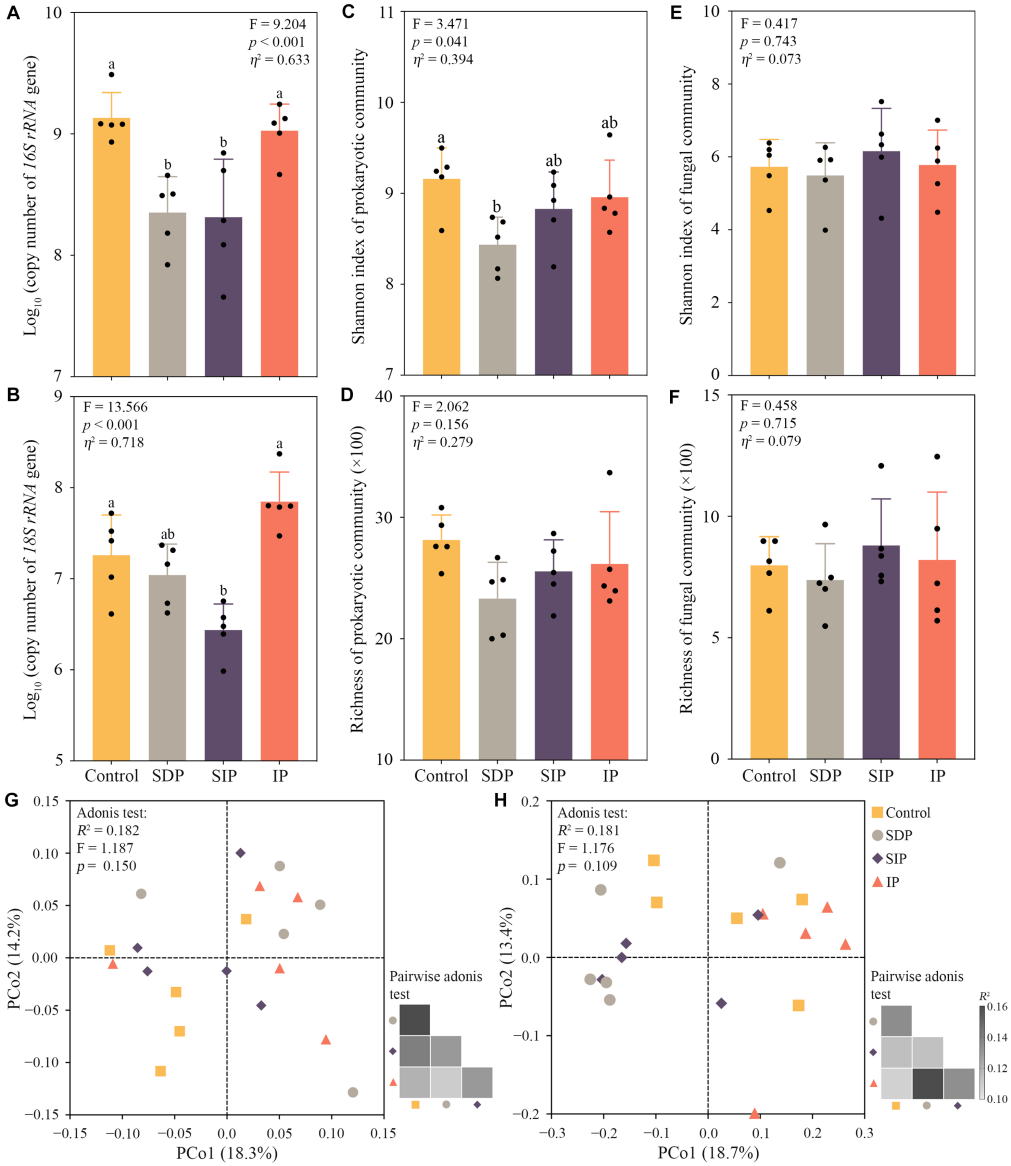
Influences of solar photovoltaic installations on soil microbial diversity and abundance in arid and semi-arid ecosystems. **(A)** Shifts in the abundance of *16S rRNA* and **(B)** *18S rRNA* gene in the soil of different treatments. Copy number means the copies of *16S rRNA* or *18S rRNA* gene per gram dry weight soil. **(C)** Variations in Shannon index and **(D)** richness of soil prokaryotic communities among treatments. **(E)** Variations in Shannon index and **(F)** richness of fungal communities across treatments. Different lowercase letters indicate significant differences at the *p* < 0.05 level. Eta squared indicated the effect size of the treatment. **(G)** Principle coordinate analysis showing changes in soil prokaryotic and **(H)** fungal community composition. Bray–Curtis distance was used in determining differences in community composition among the treatments.

The results of real-time PCR showed that the absolute abundance of the *16S rRNA* gene in SDP and SIP and that of the *18S rRNA* gene in the SIP significantly declined compared with the control fields (Figures 4A,B). The sequencing data revealed significant decline in the Shannon index of the prokaryotic community in the SDP (*p* = 0.030, Figure 4C). No apparent changes in alpha diversity indices of prokaryotic and fungal communities were observed in the control, SIP, and IP (Figures 4C–F). Similarly, the composition of soil prokaryotic (Adonis test, *R*^2^ = 0.182, *F* = 1.187, *p* = 0.150) and fungal communities (*R*^2^ = 0.181, *F* = 1.176, *p* = 0.109) showed no apparent response to solar photovoltaic panels (Figures 4G,H). Although no statistical significance was observed, differences in fungal community composition were found between the treatments of SDP/SIP and control/IP (Figure 4H), indicating the shading effect of photovoltaic panels on fungal community structure. To confirm the hypothesis, we performed the Adonis test and observed a significant shift in soil fungal community structure between the treatments with (SDP and SIP) and without the impact of shades (control and IP, *R*^2^ = 0.079, *F* = 1.534, *p* = 0.023).

Across all samples, the nine most dominant prokaryotic phyla were Actinobacteria, Proteobacteria, Firmicutes, Crenarchaeota, Chloroflexi, Acidobacteriota, Myxococcota, Bacteroidota, and Gemmatimonadota, which account for a total of 65.0–82.0% of taxon tags (Supplementary Figure S3). Meanwhile, photovoltaic panels significantly affected the proportions of Acidobacteria (Kruskal-Wallis test, *H* = 8.806, *p* = 0.032) and Myxococcota (*H* = 9.606, *p* = 0.022), which decreased by an average of 39.9 and 49.6%, respectively, in the SDP relative to those of the control (Supplementary Figure S3). Moreover, four dominant fungal phyla, including Ascomycota, Basidiomycota, Glomeromycota, and Mortierellomycota, totally accounted for 55.2–86.2% of taxon tags across all samples (Supplementary Figure S4). Statistical analysis showed that the relative abundance of Glomeromycota was significantly inhibited by shades (*H* = 12.949, *p* = 0.005, Supplementary Figure S4). On average, the relative abundance of Glomeromycota in SDP and SIP decreased to only 13.2 and 20.9% of that of the control, respectively (Supplementary Figure S4).

Variance partitioning analysis showed that photovoltaic installations (treatments), plant character, and soil abiotic property accounted for 24.1% (*F* = 1.8, *p* = 0.002) and 8.7% (*F* = 1.3, *p* = 0.006) community variance of soil prokaryotes and fungi, respectively (Supplementary Figure S5). Meanwhile, soil abiotic property independently contributed to 72.8% (adj. *R*^2^ = 17.5%, *F* = 1.7, *p* = 0.002) and 71.7% (adj. *R*^2^ = 6.3%, *F* = 1.2, *p* = 0.042) of the explanation rates for prokaryotic and fungal communities, respectively (Supplementary Figure S5). Of these soil variables, total nitrogen content caused the highest rates of changes in prokaryotic community structure (adj. *R*^2^ = 4.9%, *F* = 2.0, *p* = 0.012), and total phosphorus content was the most important variable in the fungal community (adj. *R*^2^ = 3.6%, *F* = 1.7, *p* = 0.022, Supplementary Table S3).

**Figure 5:**
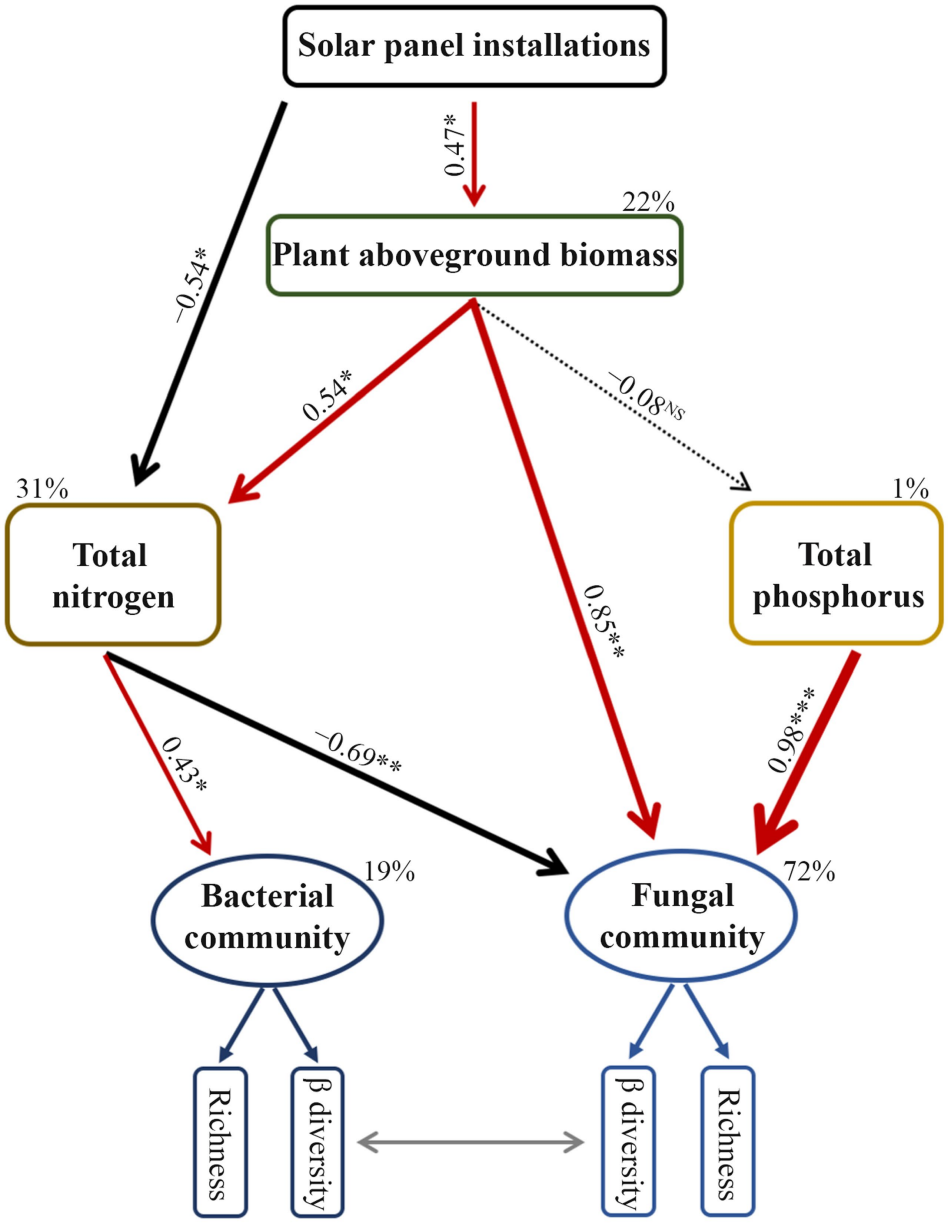
Final results of structural equation model showing the relationships among plant aboveground biomass, soil property, and prokaryotic and fungal community diversity. The model fitted well to our data (*x*^2^ = 6.070, *df* = 15, *p* = 0.979, GFI = 0.930, RMSEA < 0.001). Solid arrows indicate significant correlations; dashed arrows mean non-significant relationships; GFI, goodness-of-fit index; RMSEA, root mean squared error of approximation. The β diversity of the microbial community was quantified by using the first axis of the principal coordinate analysis. The values listed on the arrows represent standardized path coefficients. The percentage (*R*^2^) attached to the responsive factor indicates the variation rate explained by other variables. * represents *p* < 0.05; **p < 0.01; ***p < 0.001.

The structural equation model confirmed the effects of soil abiotic variables, where the content of total nitrogen and phosphorus directly explained changes in soil microbial community diversity (Figure 5). The solar photovoltaic installations directly or indirectly modified total nitrogen content by affecting plant aboveground biomass (Figure 5). Apart from the direct effect, shifts in plant aboveground biomass indirectly drove the diversity of the fungal community by altering the total nitrogen content in the soil.

### Impacts of photovoltaic panels on co-occurrence pattern of soil microbes

**Figure 6:**
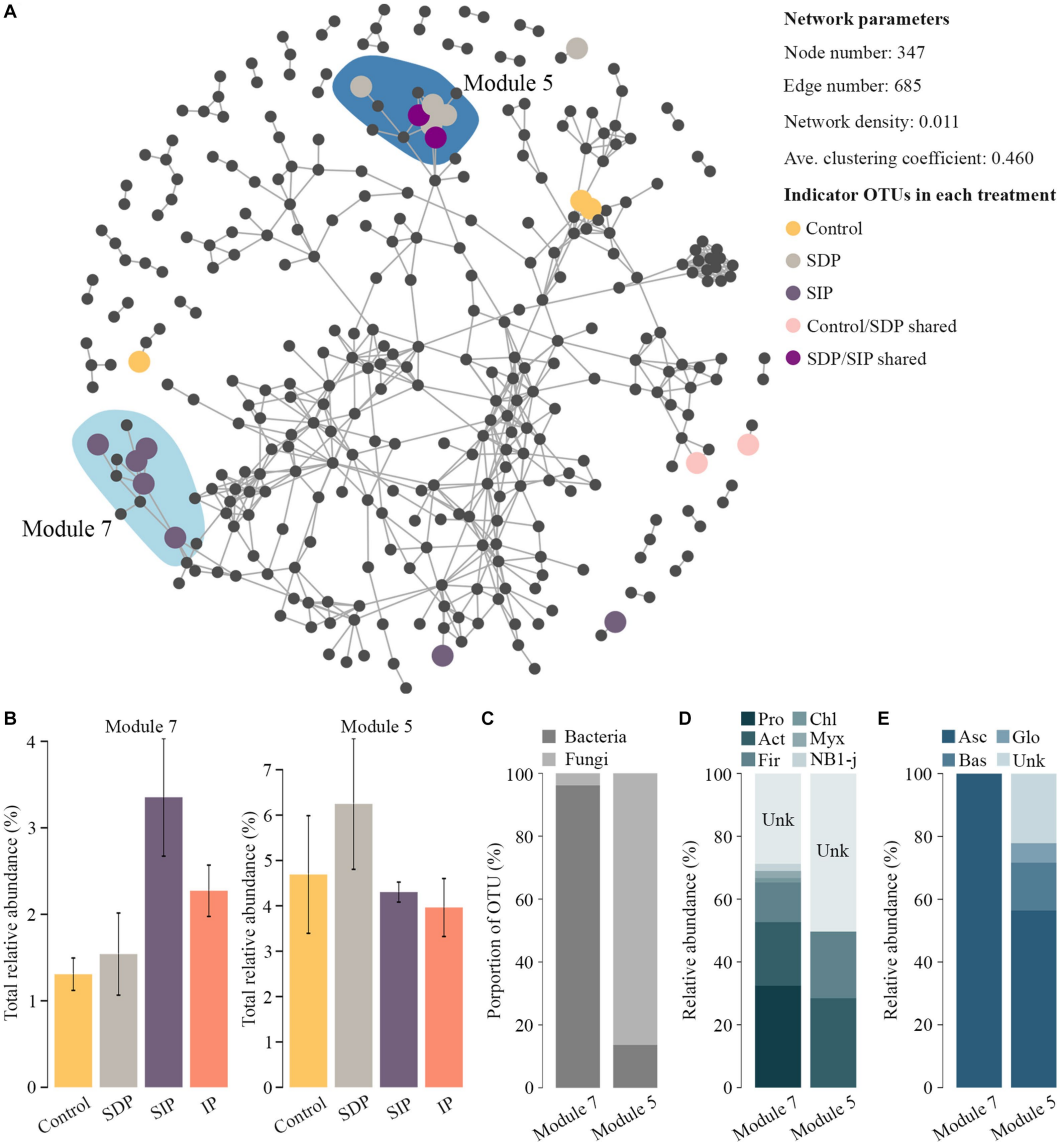
Co-occurrence patterns of treatment-specific OTUs in the soil. **(A)** Co-occurrence network showing the network modules containing treatment-specific OTUs. Indicator OTUs were colored by their association to the treatments formed by photovoltaic panels. In the shaded areas, the network modules contained high proportions of treatment-specific OTUs. **(B)** Cumulative relative abundance of soil bacterial and fungal OTUs in treatment-specific network modules. **(C)** Taxonomic composition of soil bacterial and fungal OTUs of treatment-specific network modules at the kingdom level. **(D)** Taxonomic composition of soil bacterial and **(E)** fungal OTUs of treatment-specific network modules at the phylum level. Pro represents Proteobacteria; Act, Actinobacteria; Fir, Firmicutes; Chl, Chloroflexi; Myx, Myxococcota; Asc, Ascomycota; Bas, Basidiomycota; Glo, Glomeromycota; Unk, Unknown.

To estimate the effect of photovoltaic panels on microbial co-occurrence patterns, we first constructed a co-occurrence network by combining the prokaryotic and fungal OTUs in all the samples. Using *indicator species* and *edgeR* methods, we found two treatment-specific modules, which contained indicator OTUs specific to SIP (module 7) and SDP (module 5, Figure 6A). Within each module, the OTUs’ cumulative relative abundance increased in the specific treatment (SIP or SDP) relative to that in other treatments (Figure 6B). Interestingly, the two treatment-specific modules exhibited distinct taxonomic patterns (Figure 6C). Meanwhile, module 7 was dominated by prokaryotes, which were composed of Proteobacteria, Chloroflexi, Actinobacteria, Firmicutes, Myxococcota, and NB1-j (Figure 6D), whereas module 5 mainly contained fungal OTUs, which belonged to Ascomycota, Basidiomycota, and Glomeromycota (Figure 6E).

To detect the shading effect of photovoltaic panels on co-occurrence patterns, we then constructed two separate networks according to whether the treatment was affected by shade. The results showed that the co-occurrence pattern of “with shade” treatments possessed a higher edge number, network density, average degree, and clustering coefficient than the “without shade” treatments (Supplementary Figure S6), indicating a complex microbial co-occurrence pattern in the soils under the photovoltaic panels. In general, the positive effect of shade was attributed to the increase in co-occurrences among soil prokaryotes, accounting for 53.0% of the edges increased in the “with shade” network.

## Discussion

### Solar power plants directly contribute to carbon sequestration

Apart from the positive effects on global carbon storage and reduction in the usage of fossil fuels (Turney and Fthenakis, 2011), our results showed that solar power plants would increase carbon sequestration through the direct promotion of vegetation biomass (Figure 2) and soil organic carbon content under and in front of the photovoltaic panels (Figure 3). In the treatments (2 × SDP, SIP, and IP) investigated in the present study, the average promotion rate in soil organic carbon content is 11.2%. Given that plant carbon content is about 50% of plant weight (Ma et al., 2018), carbon sequestration capacity in a solar power plant increases in the surface soil under and in front of the panels by more than 11.2% relative to that in the control field after 5-year of establishment, suggesting a positive effect of the panels on the carbon sink of arid and semi-arid ecosystems.

### Differential impacts of shading on plant diversity and vegetation biomass

Consistent with a previous study (Liu et al., 2019), the evenness and richness of plant community under the photovoltaic panels (SDP and SIP treatments) are higher than those of the control fields (Figure 1). These results suggest that in the long run, the solar power plant facilitates the recovery of vegetation diversity in the arid and semi-arid ecosystems of northwest China. In the present study, the recovery of plant diversity is mainly associated with the promoted growth of *Rtemisia frigida* and rare plant species (i.e., *Chenopodium glaucum* L., *Plantago minuta* Pall., and *Artemisia pontica* L., Supplementary Table S2). These plant species might well adapt to the shading environments, which show decreases in photosynthetic photon flux density (Wu et al., 2022) and ratio between red and far-red light (Markesteijn and Poorter, 2009).

Nevertheless, the alternation of the composition of the plant community from the *Artemisia scoparia* and *Agropyron mongolicum*-dominated community to a *Rtemisia frigida-*dominated one (Supplementary Table S2) probably increases the sensitivity of the plant community as a whole to drought stress. This inference is indirectly supported by the differential responses of plant aboveground biomass to photovoltaic panels in SDP and SIP treatments. Photovoltaic panels stimulate the plant aboveground biomass in the SIP but substantially inhibit the plant aboveground biomass in SDP by transporting rainwater from SDP to SIP (Figures 1, 2). The results suggest that in the absence of precipitation supplementation, the shading effect of the photovoltaic panels potentially strengthens the inhibitory impact of drought stress on plant growth, even though there is a slight increase in soil moisture content within the SDP treatment (Figure 3). Furthermore, the increase in belowground biomass within the SDP indirectly demonstrate the heightened drought stress experienced by plant communities (Figure 2B). Additionally, apart from the elevated drought conditions, the shades may impede plant growth in the SDP by repressing leaf water potential and photosynthetic capacity (Valladares and Pearcy, 2002). Given that the SDP accounts for approximately two-thirds of the total area occupied by the photovoltaic panel footprint, the decrease in aboveground biomass (Figure 2) and the resulting exposure of bare soil (Figure 1) may endanger the stability of arid and semi-arid ecosystems and services it provides.

### Benefits and risks of photovoltaic panels on soil microbial diversity, proliferation, and metabolic activity

Consistent with recent investigating focusing on soil archaeal community composition (Yuan et al., 2022), our findings further demonstrates the absence of conspicuous shifts in the structures of soil bacterial and fungal communities across the treatments associated with the installation of photovoltaic panels in arid regions (Figure 4). However, the panels did induced alternations in the composition of the soil fungal communities (Adonis test, *F* = 1.534, *p* = 0.023) and exhibited suppressive effects on the relative abundance of arbuscular mycorrhizal fungi (AMF) within the SDP and SIP treatments of (Supplementary Figure S4). As AMF establish symbiotic associations with the roots of a wide range of land plants (Liu et al., 2007), these results suggest that the shading effects resulting from the presence of solar panels disrupt the plant-fungus interaction in the soil, primarily mediated by the influence on plant community (Figure 2; Li et al., 2023). Moreover, there was a decline in the relative abundances of Acidobacteriota and Myxococcota within the SDP treatment (Figure 3). Considering the vital roles played by Acidobacteriota in organic matter decomposition and nutrient cycling processes (Kristensen et al., 2021), and the predator nature of Myxococcota (Zhou et al., 2014), the observed reductions in relative abundances of these microbial groups may have potential effects on soil ecosystem functioning in arid regions. These effects could potentially influence critical processes such as nutrient cycling and plant–soil-microbe interactions, despite the absence of apparent alterations in the overall composition of the microbial communities (Figure 4).

In addition to the declinations in proportions of functional microbial groups, the absolute abundance of *16S rRNA* and *18S rRNA* genes is substantially reduced in the soils under the panels as well (Figure 4), suggesting a negative effect of shades on the proliferation of soil bacterial and fungal groups. The shading effect is further influenced by the precipitation alternation resulting from the presence of the panels. Specifically, while both bacterial and fungal populations experience a decline in their reproduction under the SIP treatment, only the growth of bacteria is inhibited under the SDP treatment (Figure 4). The discrepancy implies that the variability in soil microenvironments may impose distinct growth limitations on different soil microbial populations within the two treatments.

In the treatment of SIP, photovoltaic panels increase soil moisture content nearly twofold by casting shadows and increasing precipitation in the treatment (Figure 3). As prokaryotic and fungal community composition showed no noticeable variation relative to the control fields (Figure 4), the increased soil moisture might inhibit the proliferation of soil microorganisms, which are well-adapted to dry and aerobic soil conditions. Notably, the shading effect of the panels decreases the topsoil temperature by approximately 4.0°C during the growing season in arid regions (Yue et al., 2021). This decline in temperature may directly inhibit microbial activity and growth in the soil. In addition, the increase in plant biomass observed in the SIP may intensify competition between plants and soil microbes for nutrients (i.e., phosphorus; Figure 5) and thus may constrain the growth of soil microbes in the sandy barren soils of arid and semi-arid ecosystems (Oliverio et al., 2020; Wang et al., 2020; Teste et al., 2021). These findings suggest that the combined effects of altered soil moisture, temperature, and nutrient availability due to installation of solar panels can influence the dynamics and functioning of soil microbial communities in arid ecosystems.

Compared with the SIP, the SDP treatment shows more considerable soil microenvironmental variation (particularly for prokaryotic populations) because not only the *16S rRNA* gene abundance but also the alpha diversity of prokaryotes and the activity of soil dehydrogenase are substantially inhibited (Figure 5 and Supplementary Figure S2). Given that soil dehydrogenase plays a fundamental role in the microbial oxidation of soil organic matter and accumulates intracellularly in living microbial cells (Wolińska and Stępniewska, 2012), our results suggest that the proliferation and metabolic activity of soil prokaryotes decline in the SDP treatment. As soil organic carbon content (total and dissolved) shows no change in the treatment (Figure 4), the decline in soil microbial activity is probably not related to food shortage. In contrast to the SIP, the SDP treatment shows increase in soil electrical conductivity because of reduction in plant aboveground biomass and the amount of inorganic ions absorbed by plants (Figure 3). Given that soil fungi are generally more tolerant to drought and osmotic stress than prokaryotes (Manzoni et al., 2012; Teste et al., 2021), the deprivation of precipitation by photovoltaic panels might constrain the proliferation of prokaryotic communities and microbial activities by intensifying osmotic stress or ionic toxicity or both in the soil of the SDP treatment (Setia et al., 2010; Chowdhury et al., 2011). Furthermore, our results highlight that the physiological activities of soil microbes might be more sensitive to photovoltaic installations than microbial community composition in arid and semi-arid ecosystems.

A potential positive influence of photovoltaic panels on soil microbial communities is the alteration of the microbial co-occurrence pattern (Figure 6 and Supplementary Figure S6). Either by dominating specific network modules with representative OTUs (Figure 6) or promoting the co-occurrence of OTUs (Supplementary Figure S6), photovoltaic panels markedly perplex the microbial co-occurrence pattern in the soil underneath. In line with a previous study (Hartman et al., 2018), our results show that the microbial OTUs cluster in a specific module belonging to the same kingdom (Figure 6), indicating similar responses of the microbes to the shift in soil microenvironments. A group of microorganisms with similar physiological characteristics (i.e., stress tolerance) might proliferate under environmental pressure, which increases the possibility of the co-occurrence of these microbial species in a specific niche. Even though true ecological interactions between bacteria and fungi have been partially proven (i.e., the antagonistic activity of bacteria against plant fungal pathogens; Durán et al., 2018), the influences of variations in co-occurrence patterns on the assembly and stability of soil microbial communities underneath photovoltaic panels prove to be elusive.

Notably, neither soil microbial community diversity nor physiological activity responds to the addition of precipitation in the IP treatment (Figure 4 and Supplementary Figure S2), indicating that increase in precipitation would not disturb the diversity and functionality of soil microbial communities in arid and semi-arid ecosystems. These observations are inconsistent with our previous study, which has shown that soil microbial community composition was altered by increased precipitation in the same ecosystem (Na et al., 2019b). One explanation for the inconsistency is that the timing and method for adding water and water quantity and quality (tap water or rainwater) differed between the studies. In the present study, rainwater was blocked by photovoltaic panels and then redistributed to the IP treatment. The effect of the panels enabled us to simulate the ecological effects of increased precipitation in arid and semi-arid regions in a natural way. Therefore, future simulations should consider the potential impacts of water (i.e., timing of addition and chemical composition) on the soil microbial communities.

### Potential benefits and risks of photovoltaic installations on arid and semi-arid ecosystems

According to the results of the present study, we summarized the potential benefits and risks of solar power plants in arid and semi-arid ecosystems in northwest China (Table 1). The primary positive influences of solar power plants on arid ecosystems are the stimulation of soil carbon storage and recovery of vegetation biomass and diversity (Table 1). We consider the effects of photovoltaic panels on soil microbial co-occurrence networks and community composition to be potential advantages of solar power plants. These impacts have the potential to contribute to the preservation of stability and functionality within soil microbial communities (Reed and Martiny, 2007; Guo et al., 2022). The inhibition of soil microbial physiological activity and plant aboveground biomass in specific treatments are the potential risks of the power plant in arid and semi-arid ecosystems (Table 1). Moreover, as we discussed above, a shift in the structure of the plant community under the photovoltaic panels might disturb the tolerance of plants to potential environmental stress. After systematically analyzing the results, we found that these potential negative impacts were mainly detected in the treatment of SDP (Table 1), where photovoltaic panels completely block rainwater. As such, we suppose that evenly redistributing the rainwater deposited in SIP and/or IP treatments to zones under photovoltaic panels might help diminish the potential risks of the solar power plants on arid and semi-arid ecosystems.

**Table 1:** Potential benefits and risks of solar photovoltaic installations on arid and semi-arid ecosystems.

Observation object	Benefits	Risks
Soil	Increasing soil organic carbon content	Inducing osmotic stress and/or ion toxicity in the SDP plot
Plant community	Increasing vegetation biomass in the SIP and IP plots	Decreasing aboveground biomass in the SDP plot
	Recovery of plant alpha diversity under the panels	Alternation in plant community composition under the panels
Microbial community	Perplexed microbial co-occurrence pattern	Biodiversity loss of soil prokaryotes in the SDP plot
	No obvious impact on soil microbial community structure	Inhibiting proliferation of bacteria in the SDP and SIP plots
		Inhibiting proliferation of soil fungi in the SIP plot
		Decreasing soil microbial activity in the SDP plot

Limited by constraints of conducting investigations within a solar farm, the current study provides an assessment of the potential benefits and risks associated with photovoltaic panels in arid ecosystem by comparing a control positioned between solar panel rows. However, it is essential for future study to expand upon this by examining the variations in plant and soil microbial communities between the areas with and without solar panels. Given that the composition of soil microbial communities appears unaffected by solar panels in arid ecosystems (Figure 4), future experimentations integrating metagenomic or metatranscriptomic analysis, stable isotope probing, and enzyme activity measurements could shed new light on the functional genes and pathways influenced by solar panels. Gaining insights into these functional shifts in the present or absence of photovoltaic panels will contribute to a more comprehensive understanding of the ecological impacts of solar farms on vital ecosystem processes in arid regions.

## Conclusion

In conclusion, our study provides valuable insights into the potential benefits and risks associated with solar power plants in arid and semi-arid ecosystems of northwest China. The presence of solar power plants has the potential to stimulate soil carbon storage, promote the recovery of plant community diversity, and positively influence soil microbial co-occurrence network. However, there are also potential risks associated with solar farms, including the inhibition of soil microbial proliferation and metabolic activity, as well as a decrease in plant aboveground biomass, particularly in areas affected by shading and precipitation-alternation effects. The observed risks primarily arouse from the reduction in precipitation. We propose the redistribution of rainwater to the area underneath photovoltaic panels as a strategy to help mitigate the potential negative impacts on dryland ecosystems. Our findings highlight the complex interactions between solar panels, plants, and soil microbial communities, underscoring the need for sustainable practices in solar energy development to minimize potential ecological consequences.

## Data availability statement

The datasets presented in this study can be found in online repositories. The names of the repository/repositories and accession number(s) can be found at: https://dataview.ncbi.nlm.nih.gov/object/PRJNA883931, PRJNA883931.

## Author contributions

ZL: sampling, data collection, and writing. TP: data collection and analysis. SM: data analysis. CQ: sampling. YS: data curation. CZ, KL, NG, and MP: data collection. XW: writing. YB: supervision. XN: conceptualization, methodology, sampling, writing—original draft, and writing review and editing. All authors contributed to the article and approved the submitted version.

## Funding

This work was supported by grants from the National Natural Science Foundation of China (Nos. 32271698 and 31760612).

## Conflict of interest

The authors declare that the research was conducted in the absence of any commercial or financial relationships that could be construed as a potential conflict of interest.

## Publisher’s note

All claims expressed in this article are solely those of the authors and do not necessarily represent those of their affiliated organizations, or those of the publisher, the editors and the reviewers. Any product that may be evaluated in this article, or claim that may be made by its manufacturer, is not guaranteed or endorsed by the publisher.

## References

[ref1] Banerjee S. Helgason B. Wang L. Winsley T. Ferrari B. C. Siciliano S. D. (2016). Legacy effects of soil moisture on microbial community structure and N_2_O emissions. Soil Biol. Biochem. 95, 40–50. doi: 10.1016/j.soilbio.2015.12.004

[ref2] Barron-Gafford G. A. Minor R. L. Allen N. A. Cronin A. D. Brooks A. E. Pavao-Zuckerman M. A. (2016). The photovoltaic heat island effect: larger solar power plants increase local temperatures. Sci. Rep. 6, 1–7. doi: 10.1038/srep35070 27733772 PMC5062079

[ref3] Bell C. McIntyre N. Cox S. Tissue D. Zak J. (2008). Soil microbial responses to temporal variations of moisture and temperature in a Chihuahuan Desert grassland. Microb. Ecol. 56, 153–167. doi: 10.1007/s00248-007-9333-z, PMID: 18246293

[ref4] Bell C. W. Tissue D. T. Loik M. E. Wallenstein M. D. Acosta-Martinez V. Erickson R. A.. (2014). Soil microbial and nutrient responses to 7 years of seasonally altered precipitation in a Chihuahuan Desert grassland. Glob. Chang. Biol. 20, 1657–1673. doi: 10.1111/gcb.12418, PMID: 24115607

[ref5] Borneman J. Hartin R. J. (2000). PCR primers that amplify fungal rRNA genes from environmental samples. Appl. Environ. Microbiol. 66, 4356–4360. doi: 10.1128/AEM.66.10.4356-4360.2000, PMID: 11010882 PMC92308

[ref6] Caporaso J. G. Kuczynski J. Stombaugh J. Bittinger K. Bushman F. D. Costello E. K.. (2010). QIIME allows analysis of highthroughput community sequencing data. Nat. Methods 7, 335–336. doi: 10.1038/nmeth.f.303, PMID: 20383131 PMC3156573

[ref7] Chowdhury N. Marschner P. Burns R. G. (2011). Soil microbial activity and community composition: impact of changes in matric and osmotic potential. Soil Biol. Biochem. 43, 1229–1236. doi: 10.1016/j.soilbio.2011.02.012

[ref8] Coutts A. M. Tapper N. J. Beringer J. Loughnan M. Demuzere M. (2013). Watering our cities: the capacity for water sensitive urban design to support urban cooling and improve human thermal comfort in the Australian context. Prog. Phys. Geogr. 37, 2–28. doi: 10.1177/0309133312461032

[ref9] Dick W. A. (2011). “Development of a soil enzyme reaction assay (Chapter 4)” in Methods of soil enzymology. ed. Dick R. P. (Madison, WI, USA: ASA-CSSA-SSSA Book and Multimedia Publishing), 71–84.

[ref10] Durán P. Thiergart T. Garrido-Oter R. Agler M. Kemen E. Schulze-Lefert P.. (2018). Microbial interkingdom interactions in roots promote Arabidopsis survival. Cells 175, 973–983.e14. doi: 10.1016/j.cell.2018.10.020, PMID: 30388454 PMC6218654

[ref01] Edgar R. C. (2013). UPARSE: highly accurate OTU sequences from microbial amplicon reads. Nat. Methods 10, 996–998. doi: 10.1038/nmeth.2604 23955772

[ref11] Fierer N. Jackson J. A. Vilgalys R. Jackson R. B. (2005). Assessment of soil microbial community structure by use of taxon-specific quantitative PCR assays. Appl. Environ. Microbiol. 71, 4117–4120. doi: 10.1128/AEM.71.7.4117-4120.2005, PMID: 16000830 PMC1169028

[ref12] Frankeberger W. T. Johanson J. B. (1983). Method of measuring invertase activity in soils. Plant Soil 74, 301–311. doi: 10.1007/BF02181348

[ref13] Guo B. Zhang L. Sun H. Gao M. Yu N. Zhang Q.. (2022). Microbial co-occurrence network topological properties link with reactor parameters and reveal importance of low-abundance genera. NPJ Biofilms Microbiomes 8, 1–13. doi: 10.1038/s41522-021-00263-y 35039527 PMC8764041

[ref14] Hartman K. van der Heijden M. G. Wittwer R. A. Banerjee S. Walser J. C. Schlaeppi K. (2018). Cropping practices manipulate abundance patterns of root and soil microbiome members paving the way to smart farming. Microbiome 6:14. doi: 10.1186/s40168-017-0389-9 29338764 PMC5771023

[ref15] Hassanpour Adeh E. Selker J. S. Higgins C. W. (2018). Remarkable agrivoltaic influence on soil moisture, micrometeorology and water-use efficiency. PLoS One 13:e0203256. doi: 10.1371/journal.pone.0203256, PMID: 30383761 PMC6211631

[ref16] He C. Kim H. Hashizume M. Lee W. Honda Y. Kim S. E.. (2022). The effects of night-time warming on mortality burden under future climate change scenarios: a modelling study. Lancet Planetary Health 6, e648–e657. doi: 10.1016/S2542-5196(22)00139-5, PMID: 35932785

[ref17] Hernandez R. R. Tanner K. E. Haji S. Parker I. M. Pavlik B. M. Moore-O’Leary K. A. (2020). Simulated photovoltaic solar panels alter the seed bank survival of two desert annual plant species. Plan. Theory 9:1125. doi: 10.3390/plants9091125, PMID: 32878043 PMC7570262

[ref18] Kandeler E. Luxhøi J. Tscherko D. Magid J. (1999). Xylanase, invertase and protease at the soil–litter interface of a loamy sand. Soil Biol. Biochem. 31, 1171–1179. doi: 10.1016/S0038-0717(99)00035-8

[ref19] Kõljalg U. Nilsson R. H. Abarenkov K. Tedersoo L. Taylor A. F. Bahram M.. (2013). Towards a unified paradigm for sequence-based identification of fungi. Mol. Ecol. 22, 5271–5277. doi: 10.1111/mec.12481, PMID: 24112409

[ref20] Kristensen J. M. Singleton C. Clegg L. A. Petriglieri F. Nielsen P. H. (2021). High diversity and functional potential of undescribed “Acidobacteriota” in Danish wastewater treatment plants. Front. Microbiol. 12:643950. doi: 10.3389/fmicb.2021.643950, PMID: 33967982 PMC8100337

[ref21] Kumar S. Chaudhuri S. Maiti S. K. (2013). Soil dehydrogenase enzyme activity in natural and mine soil-a review. Middle East J. Sci. Res. 13, 898–906. doi: 10.5829/idosi.mejsr.2013.13.7.2801

[ref22] Li Y. Kalnay E. Motesharrei S. Rivas J. Kucharski F. Kirk-Davidoff D.. (2018). Climate model shows large-scale wind and solar farms in the Sahara increase rain and vegetation. Science 361, 1019–1022. doi: 10.1126/science.aar5629, PMID: 30190404

[ref23] Li C. Liu J. Bao J. Wu T. Chai B. (2023). Effect of light heterogeneity caused by photovoltaic panels on the plant–soil–microbial system in solar park. Land 12:367. doi: 10.3390/land12020367

[ref24] Liu J. Wu L. Wei S. Xiao X. Su C. Jiang P.. (2007). Effects of arbuscular mycorrhizal fungi on the growth, nutrient uptake and glycyrrhizin production of licorice (*Glycyrrhiza uralensis* Fisch). Plant Growth Regul. 52, 29–39. doi: 10.1007/s10725-007-9174-2

[ref25] Liu Y. Zhang R. Q. Huang Z. Cheng Z. López-Vicente M. Ma X. R.. (2019). Solar photovoltaic panels significantly promote vegetation recovery by modifying the soil surface microhabitats in an arid sandy ecosystem. Land Degrad. Dev. 30, 2177–2186. doi: 10.1002/ldr.3408

[ref26] Liu Y. Zhang R. Q. Ma X. R. Wu G. L. (2020). Combined ecological and economic benefits of the solar photovoltaic industry in arid sandy ecosystems. J. Clean. Prod. 262:121376. doi: 10.1016/j.jclepro.2020.121376

[ref27] Liu W. Zhang Z. Wan S. (2009). Predominant role of water in regulating soil and microbial respiration and their responses to climate change in a semiarid grassland. Glob. Chang. Biol. 15, 184–195. doi: 10.1111/j.1365-2486.2008.01728.x

[ref28] Ma S. He F. Tian D. Zou D. Yan Z. Yang Y.. (2018). Variations and determinants of carbon content in plants: a global synthesis. Biogeosciences 15, 693–702. doi: 10.5194/bg-15-693-2018

[ref29] Magoč T. Salzberg S. L. (2011). FLASH: fast length adjustment of short reads to improve genome assemblies. Bioinformatics 27, 2957–2963. doi: 10.1093/bioinformatics/btr507, PMID: 21903629 PMC3198573

[ref30] Manzoni S. Schimel J. P. Porporato A. (2012). Responses of soil microbial communities to water stress: results from a meta-analysis. Ecology 93, 930–938. doi: 10.1890/11-0026.1, PMID: 22690643

[ref31] Markesteijn L. Poorter L. (2009). Seedling root morphology and biomass allocation of 62 tropical tree species in relation to drought-and shade-tolerance. J. Ecol. 97, 311–325. doi: 10.1111/j.1365-2745.2008.01466.x

[ref32] McCrary M.D. McKernan R.L. Flanagan P.A. Wagner W.D. (1984). Wildlife interactions at solar one. Final report (No. DOE/SF/10501-306; STMPO-606). Los Angeles County Natural History Museum Foundation, CA (USA). Section of Ornithology.

[ref33] Na X. Ma C. Ma S. Ma X. Zhu X. Xu P.. (2019b). Monocropping decouples plant–bacteria interaction and strengthens phytopathogenic fungi colonization in the rhizosphere of a perennial plant species. Plant Soil 445, 549–564. doi: 10.1007/s11104-019-04311-7

[ref34] Na X. Yu H. Wang P. Zhu W. Niu Y. Huang J. (2019a). Vegetation biomass and soil moisture coregulate bacterial community succession under altered precipitation regimes in a desert steppe in northwestern China. Soil Biol. Biochem. 136:107520. doi: 10.1016/j.soilbio.2019.107520

[ref35] Nielsen U. N. Ball B. A. (2015). Impacts of altered precipitation regimes on soil communities and biogeochemistry in arid and semi-arid ecosystems. Glob. Chang. Biol. 21, 1407–1421. doi: 10.1111/gcb.12789, PMID: 25363193

[ref36] Oliverio A. M. Bissett A. McGuire K. Saltonstall K. Turner B. L. Fierer N. (2020). The role of phosphorus limitation in shaping soil bacterial communities and their metabolic capabilities. mBio 11, e01718–e01720. doi: 10.1128/mBio.01718-20 33109755 PMC7593963

[ref37] Olsen S.R. Cole C.V. Watanabe F.S. Dean L.A. (1954) Estimation of available phosphorus in soils by extraction with sodium bicarbonate. USDA Circular No. 939. U.S. Department of Agriculture, Washington, DC, pp. 19.

[ref38] Pu Y. Wang P. Wang Y. Qiao W. Wang L. Zhang Y. (2021). Environmental effects evaluation of photovoltaic power industry in China on life cycle assessment. J. Clean. Prod. 278:123993. doi: 10.1016/j.jclepro.2020.123993

[ref39] Qiu T. Wang L. Lu Y. Zhang M. Qin W. Wang S.. (2022). Potential assessment of photovoltaic power generation in China. Renew. Sust. Energ. Rev. 154:111900. doi: 10.1016/j.rser.2021.111900

[ref40] Quast C. Pruesse E. Yilmaz P. Gerken J. Schweer T. Yarza P.. (2012). The SILVA ribosomal RNA gene database project: improved data processing and web-based tools. Nucleic Acids Res. 41, D590–D596. doi: 10.1093/nar/gks1219, PMID: 23193283 PMC3531112

[ref41] Reed H. E. Martiny J. B. (2007). Testing the functional significance of microbial composition in natural communities. FEMS Microbiol. Ecol. 62, 161–170. doi: 10.1111/j.1574-6941.2007.00386.x 17937673

[ref42] Setia R. Marschner P. Baldock J. Chittleborough D. (2010). Is CO_2_ evolution in saline soils affected by an osmotic effect and calcium carbonate? Biol. Fertil. Soils 46, 781–792. doi: 10.1007/s00374-010-0479-3

[ref43] Strukelj M. Parker W. Corcket E. Augusto L. Khlifa R. Jactel H.. (2021). Tree species richness and water availability interact to affect soil microbial processes. Soil Biol. Biochem. 155:108180. doi: 10.1016/j.soilbio.2021.108180

[ref44] Sun Y. Chen S. Xie L. Hong R. Shen H. (2014). (2014) investigating the impact of shading effect on the characteristics of a large-scale grid-connected PV power plant in Northwest China. Int. J. Photoenergy 2014, 1–9. doi: 10.1155/2014/763106

[ref45] Szilágyi A. Gróf G. (2020). Estimating the environmental footprint of a grid-connected 20 MWp photovoltaic system. Sol. Energy 197, 491–497. doi: 10.1016/j.solener.2020.01.028

[ref46] Teste F. P. Lambers H. Enowashu E. E. Laliberté E. Marhan S. Kandeler E. (2021). Soil microbial communities are driven by the declining availability of cations and phosphorus during ecosystem retrogression. Soil Biol. Biochem. 163:108430. doi: 10.1016/j.soilbio.2021.108430

[ref47] Turney D. Fthenakis V. (2011). Environmental impacts from the installation and operation of large-scale solar power plants. Renew. Sust. Energ. Rev. 15, 3261–3270. doi: 10.1016/j.rser.2011.04.023

[ref48] Valladares F. Pearcy R. W. (2002). Drought can be more critical in the shade than in the sun: a field study of carbon gain and photo-inhibition in a Californian shrub during a dry El Niño year. Plant Cell Environ. 25, 749–759. doi: 10.1046/j.1365-3040.2002.00856.x

[ref49] Wang B. Huang S. Li Z. Zhou Z. Huang J. Yu H.. (2022). Factors driving the assembly of prokaryotic communities in bulk soil and rhizosphere of *Torreya grandis* along a 900-year age gradient. Sci. Total Environ. 837:155573. doi: 10.1016/j.scitotenv.2022.155573, PMID: 35504392

[ref50] Wang Z. Yang S. Wang R. Xu Z. Feng K. Feng X.. (2020). Compositional and functional responses of soil microbial communities to long-term nitrogen and phosphorus addition in a calcareous grassland. Pedobiologia 78:150612. doi: 10.1016/j.pedobi.2019.150612

[ref51] Wolińska A. Stępniewska Z. (2012). Dehydrogenase activity in the soil environment. Dehydrogenases 10, 183–210. doi: 10.5772/48294

[ref52] Wu C. Liu H. Yu Y. Zhao W. Liu J. Yu H.. (2022). Ecohydrological effects of photovoltaic solar farms on soil microclimates and moisture regimes in arid Northwest China: a modeling study. Sci. Total Environ. 802:149946. doi: 10.1016/j.scitotenv.2021.149946, PMID: 34525759

[ref53] Xia Z. Li Y. Chen R. Sengupta D. Guo X. Xiong B.. (2022). Mapping the rapid development of photovoltaic power stations in northwestern China using remote sensing. Energy Rep. 8, 4117–4127. doi: 10.1016/j.egyr.2022.03.039

[ref54] Yang L. Gao X. Lv F. Hui X. Ma L. Hou X. (2017). Study on the local climatic effects of large photovoltaic solar farms in desert areas. Sol. Energy 144, 244–253. doi: 10.1016/j.solener.2017.01.015

[ref55] Yuan B. Wu W. Yue S. Zou P. Yang R. Zhou X. (2022). Community structure, distribution pattern, and influencing factors of soil Archaea in the construction area of a large-scale photovoltaic power station. Int. Microbiol. 25, 571–586. doi: 10.1007/s10123-022-00244-x, PMID: 35347497

[ref56] Yue S. Guo M. Zou P. Wu W. Zhou X. (2021). Effects of photovoltaic panels on soil temperature and moisture in desert areas. Environ. Sci. Pollut. Res. 28, 17506–17518. doi: 10.1007/s11356-020-11742-8, PMID: 33400111

[ref57] Zhou X. W. Li S. G. Li W. Jiang D. M. Han K. Wu Z. H.. (2014). Myxobacterial community is a predominant and highly diverse bacterial group in soil niches. Environ. Microbiol. Rep. 6, 45–56. doi: 10.1111/1758-2229.12107, PMID: 24596262

