# Supplementary material for: Potential benefits and risks of solar photovoltaic power plants on arid and semi-arid ecosystems: an assessment of soil microbial and plant communities
Source: Front Microbiol. 2023 Aug 1;14:1190650. doi: 10.3389/fmicb.2023.1190650 (PMC10427150; doi:10.3389/fmicb.2023.1190650)
Supplement: Supplementary file 1 [file Data_Sheet_1.pdf]

Supplementary Material

Potential benefits and risks of solar photovoltaic power plants on the arid and semi-arid ecosystems: An assessment of soil microbial and plant communities

Ziyu Liu[a*], Tong Peng[a*], Shaolan Ma[b], Chang Qi[a], Yanfang Song[a], Chuanji Zhang[a], Kaile Li[a], Na Gao[a], Meiyun Pu[a], Xiaomin Wang[a], Yurong Bi[a#], Xiaofan Na[a#]

[a] Key Laboratory of Cell Activities and Stress Adaptations, Ministry of Education, School of Life Sciences, Lanzhou University, Lanzhou 730000, China

[b] No.1 Middle School of Pengyang, Guyuan 756000, China

[*] These authors contribute equally to this work

[#]Corresponding authors:

Xiaofan Na

Tel/Fax: +86-931-8911781

E-mail: naxf@lzu.edu.cn

Yurong Bi

Tel/Fax: +86-931-8911781

E-mail: yrbi@lzu.edu.cn

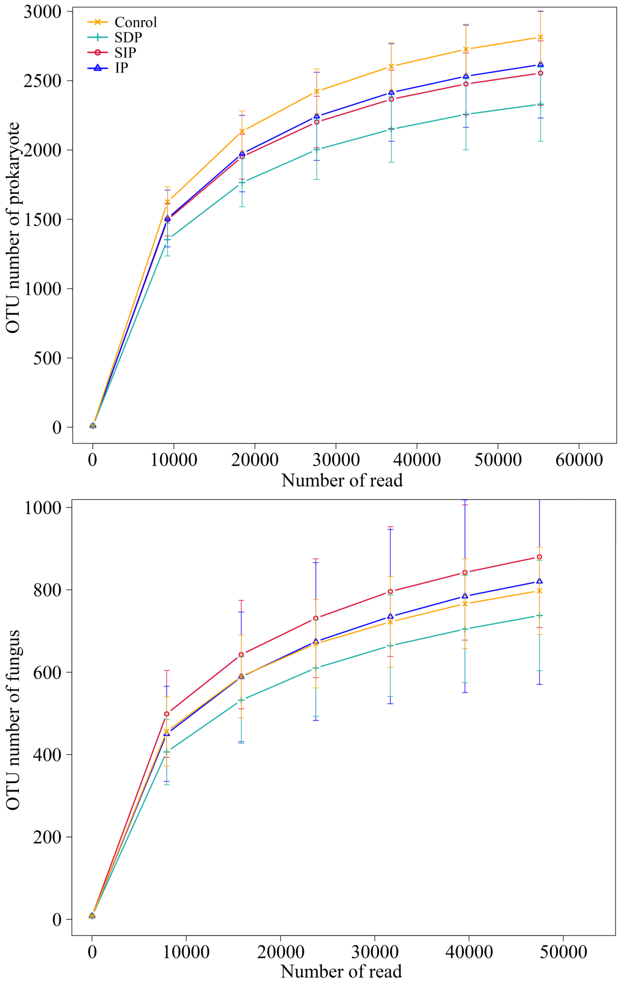

Fig. S1 Rarefaction curves of the prokaryotic and fungal communities with increasing read number.

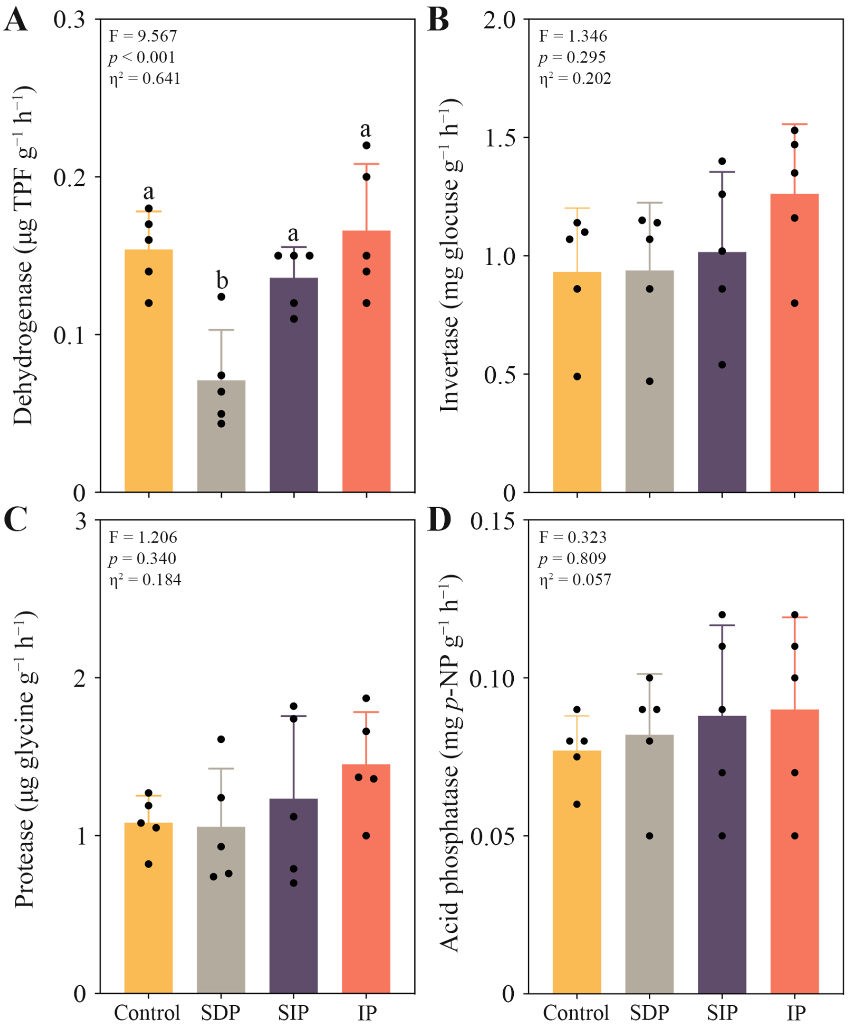

Fig. S2 Impacts of solar photovoltaic panels on the soil enzyme activities in arid and semi-arid ecosystems. Different lowercase letters indicate significant differences at a *p* of <0.05 level. Eta squared indicates the effect size of the treatment.

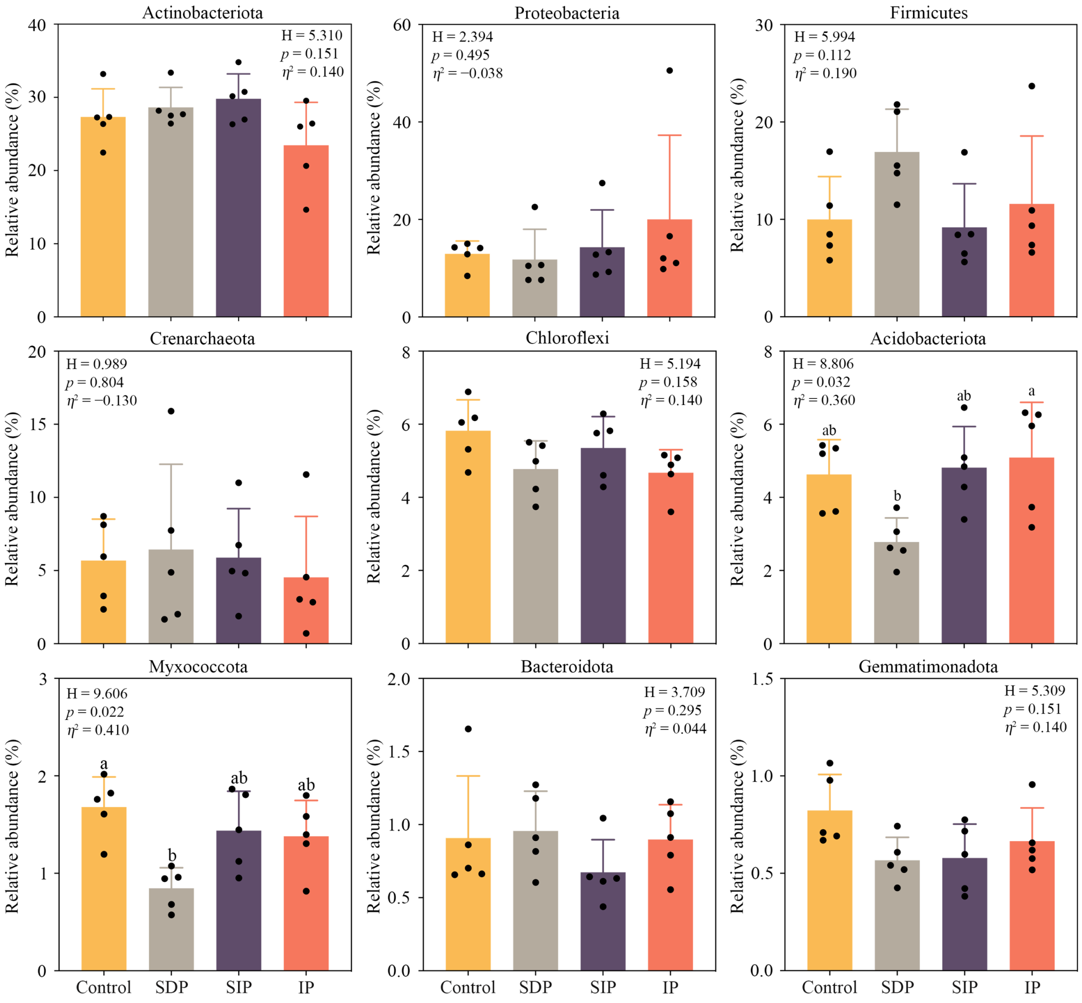

Fig. S3 Variations in the relative abundances of dominant prokaryotic phyla in the soils of distinct plots formed by solar photovoltaic panels. Different lowercase letters indicate significant differences at the $p < 0.05$ level. The effect size of the treatment was quantified by using Eta squared.

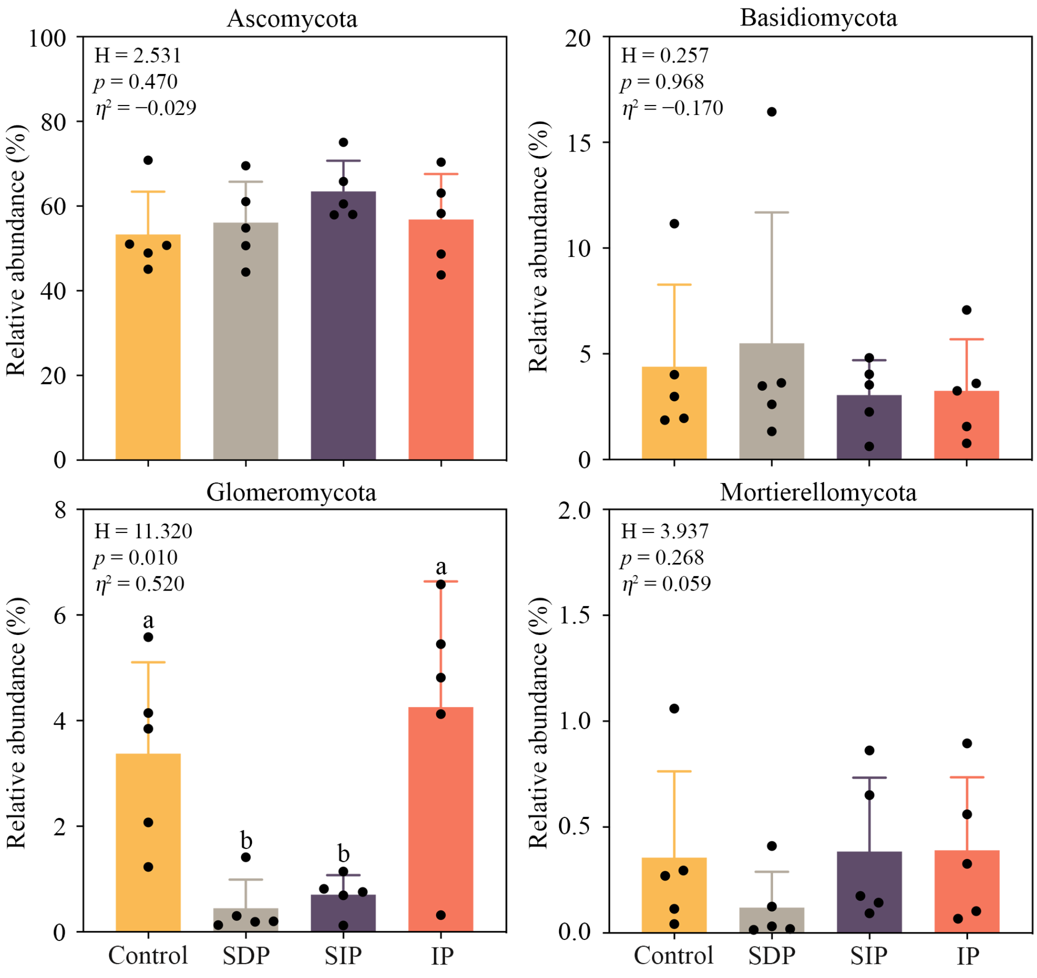

Fig. S4 Variations in the relative abundances of dominant fungal phyla in the soils of distinct plots. Different lowercase letters indicate significant differences at the $p < 0.05$ level. The effect size of the treatment was quantified by using Eta squared.

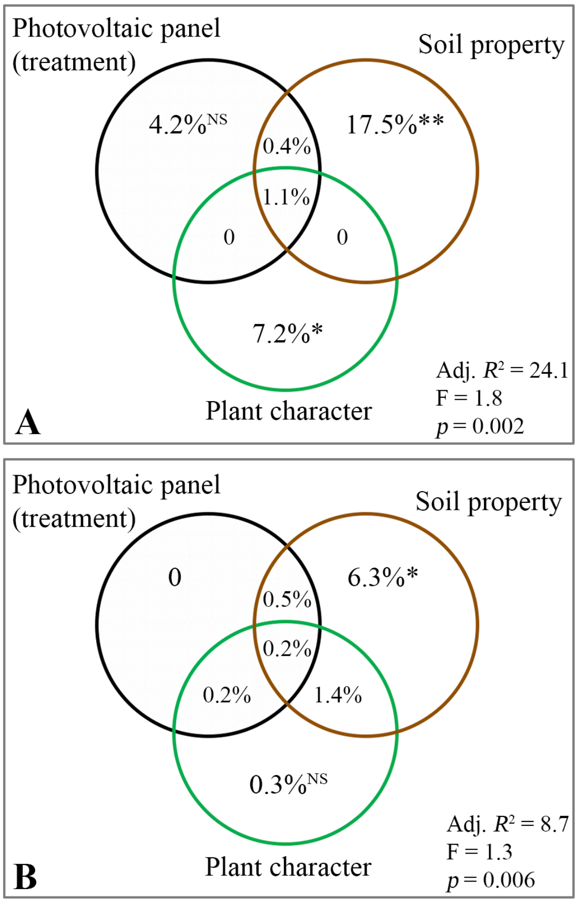

Fig. S5 Variance partitioning analysis showing the explanation rates of different data categories on the community variance of (A) prokaryotes and (B) fungi. The variables of each data category were first selected by using a forward selection protocol. In a prokaryotic community, soil property includes total nitrogen and phosphorus content, available phosphorus concentration, dissolved organic carbon content, and gravitropic water content; plant character contains plant community richness and aboveground biomass. As for soil fungal community, soil property includes total phosphorus and organic carbon content, pH, total nitrogen content, and electrical conductivity, and plant character includes the Shannon index of plant community. An overlap indicates the interactive effect of different data categories. NS represents no significant effect; *, $p < 0.05$; **, $p < 0.01$.

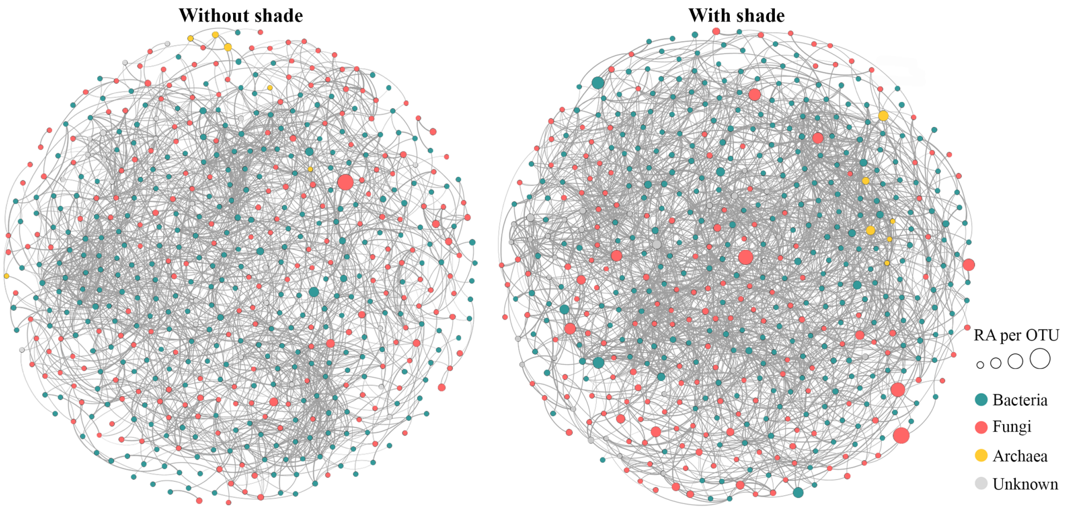

Parameters of co-occurrance networks

Network parameters	Control	Shadow
Number of node	550	539
Number of edge	2191	2668
Network density	0.015	0.018
Average degree	7.967	9.900
Average path length	4.293	3.895
Average clustering coefficient	0.309	0.329
Percentage of copersence	62.6%	63.4%
Percentage of mutual exclusion	37.4%	36.6%

Fig. S6 Variations in co-occurrence pattern of soil microbes under the photovoltaic panels. The "Without shade" network included the plots of control and IP (*n* = 10); the "With shade" network contained the plots of SDP and SIP under solar photovoltaic panels (*n* = 10). Visualization of the network was done with Gephi (v 0.9.3).

Table S1 Quality of the amplicon sequencing performed in the present study.

	Mean taxon tag	Mean OTU number	Average length (nt)	QC30	Effective (%)
Prokaryote	59,123	2,815	418	94.6	75.5
Fungus	65,178	906	317	96.4	87.3

Table S2 Impacts of photovoltaic panels on the relative abundance of dominant plant species in arid and semi-arid ecosystems.

**Plant species**	**Control**	**SDP**	**SIP**	**IP**	**Effect size**
Artemisia scoparia	33.5 ± 24.4ab	13.5 ± 20.9b	23.5 ± 11.7ab	44.8 ± 13.7a	0.14
Agropyron mongolicum	41.5 ± 24.9a	15.4 ± 15.8ab	5.2 ± 29.2b	34.6 ± 24.9a	0.32
Rtemisia frigida Willd.	1.3 ± 1.7b	18.6 ± 1.8a	26.4 ± 29.9a	0.1 ± 4.2b	0.38
Stipa capillata L.	8.4 ± 11.9	15.0 ± 4.7	8.3 ± 14.8	10.3 ± 14.8	−0.15
Other	15.3 ± 10.0bc	37.3 ± 16.2a	36.5 ± 11.8ab	10.1 ± 13.5c	0.33

Note: The relative abundance means the proportion of individual plant species' aboveground biomass accounting for the total aboveground biomass in each plot. Effect size was calculated by using Eta squared.

Table S3 Distance based RDA analysis determining the independent effect of selected soil abiotic property on the shift in community structure of soil microbes in arid and semi-arid ecosystems.

**Community**	**Variable**	**adj. R^2**	**F**	***p* value**
Prokaryotic community	**All variables**	**15.6**	**1.7**	**0.002**
	Total nitrogen	4.9	2.0	0.012
	Dissolved organic carbon	2.9	1.6	0.042
	Total phosphorus	2.6	1.5	0.062
	Gravitropic water content	2.4	1.5	0.074
	Available phosphorus	2.3	1.4	0.082
Fungal community	**All variables**	**10.5**	**1.4**	**0.002**
	Total phosphorus	3.6	1.7	0.022
	Total organic carbon	2.5	1.5	0.058
	Electrical conductivity	1.2	1.2	0.170
	pH	0.9	1.2	0.250
	Total nitrogen	0.0	0.9	0.622
